# The Cockroach: Its Destruction and Dispersal

**Published:** 1918-01

**Authors:** 


***The Cockroach : Its Destruction and Dispersal.*** (A Comparison of Insecticides and Methods.) By J. J. B. Holt, Ch. B. Vict. Abs. from the *Lancet*, June 3, 1917.

H. records the results of extensive experimentation to discover a means of combating the cockroach pest. He tested about 90 different volatile bodies, aromatic oils, coal-tar derivatives, paraffin derivatives, and dusting powders; also, about 10 food poisons. He believes that the results of these tests may hold as good for other insects as for the cockroach. Of the latter he notes that it has extraordinary powers of endurance, and that its weakest point of attack is its respiration, whereas food poisons, such as arsenic and phosphorus, have little effect upon it. H. believes that when poisons cause the cockroach to disappear, they do so because of their fumes.

A number of bodies kill the cockroach quickly through respiration. He recommends the use of bromine and sulphur dioxide (from burning sulphur) as the most efficient destroyers when mere expulsion of the insect is not sufficient. The sulphur is much cheaper. The rooms treated should be sealed. For domestic use, where respiratory irritants are sufficient, he recommends wood naphtha, an aromatic oil, or creosote. He states, however, that daily renewal is necessary for a considerable period, at least two or three weeks. He finds paraffin derivatives very effective, but does not recommend their use because of danger from fire. He thinks them superior to coal-tar products.

Of the aromatic oils the author finds rosemary, eucalyptus, and citronella the most effective in the order named, not only for the cockroach but also for the mosquito. These may easily be used on the clothing.

Of the dusting powders the author finds sodium fluoride more effective that Dalmatian or Keating powder.

Although he has not applied these tests thoroughly to any insect except the cockroach, he thinks they would produce the same results with small insects.

